# The fitness costs of antibiotic resistance mutations

**DOI:** 10.1111/eva.12196

**Published:** 2014-08-27

**Authors:** Anita H Melnyk, Alex Wong, Rees Kassen

**Affiliations:** 1 Centre for Advanced Research in Environmental Genomics, Department of Biology, University of OttawaOttawa, ON, Canada; 2 Department of Biology, Carleton UniversityOttawa, ON, Canada

**Keywords:** adaptation, antibiotic resistance, fitness

## Abstract

Antibiotic resistance is increasing in pathogenic microbial populations and is thus a major threat to public health. The fate of a resistance mutation in pathogen populations is determined in part by its fitness. Mutations that suffer little or no fitness cost are more likely to persist in the absence of antibiotic treatment. In this review, we performed a meta-analysis to investigate the fitness costs associated with single mutational events that confer resistance. Generally, these mutations were costly, although several drug classes and species of bacteria on average did not show a cost. Further investigations into the rate and fitness values of compensatory mutations that alleviate the costs of resistance will help us to better understand both the emergence and management of antibiotic resistance in clinical settings.

## Introduction

The initial optimism accompanying the introduction of antibiotics to control infection over 60 years ago has been steadily worn down by continuing reports of antimicrobial resistance (AMR) among nearly all human-associated pathogens (Palumbi 2001; Perron et al. 2006). AMR already represents a major burden on healthcare systems around the world. Estimates of the economic burden of AMR are estimated to be at least 1.5 billion euros annually in Europe (World Health Organization 2012) and on the order of $200 million annually in Canada alone (Conly 2002), and these costs are expected to get worse with time.

Widespread therapeutic and prophylactic use of antibiotics in health care and agriculture constitutes a strong and persistent selective pressure favoring the evolution of antibiotic-resistant strains, a phenomenon characterized by Hall (2004) as ‘use it and lose it’. For this reason, research has been increasingly focused on eliminating, or at least controlling, AMR once it has evolved. The most common strategy is to stop using antibiotics, the assumption being that mutations conferring resistance impose a large fitness cost in the absence of the drug. Note that fitness is taken here to be the rate of replication under prevailing environmental conditions and can be measured through competitive fitness trials or as the growth rate of the strain or population being considered. Sensitive genotypes that do not pay a cost of resistance should therefore replace resistant strains at a rate proportional to the magnitude of the cost imposed by resistance (Levin et al. 1997; Johnsen et al. 2009).

Resistance mutations may be expected to impart a fitness cost because they target important biological functions in the cell (Table 1). For example, resistance to fluoroquinolones in pseudomonads can cause impaired motility (Stickland et al. 2010), and resistance to aminoglycosides can alter the structure of the ribosome (Springer et al. 2001; Holberger and Hayes 2009) and so interfere with basic cellular functions.

**Table 1:** Antibiotics included in this meta-analysis

Antibiotic class	Examples of antibiotics (included in this study)	Mode of action (Target)	Mechanisms of resistance	Known genes involved in mutations conferring resistance
Alpha-pyrone	Myxopyronin	RNA replication: inhibits bacterial RNA polymerase (RNAP)	Altered target	*rpoB*, *rpoC*
Aminoglycoside	Amikacin, streptomycin, spectinomycin	Protein synthesis: binds to 30S subunit bacterial ribosome inhibiting translation	Drug efflux, altered target, enzymatic inhibition of drug	*rpsL*, *rrs*, *rrl, rpsE*
Coumarin	Coumermycin, novobiocin	DNA replication: inhibits DNA gyrase and topoisomerase IV enzyme	Drug efflux, altered target	*gyrB*
Dihydrofolate reductase inhibitor	Trimethoprim	DNA replication: blocks the folate coenzyme biosynthetic pathway, essential for providing monomers for DNA synthesis	Decrease thymidine requirement, altered target	*dfrA*
Fusidane	Fusidic acid	Protein synthesis: prevents the turnover of elongation factor G from the ribosome	Drug efflux, mutations in elongation factor G	*fusA*
Macrolide	Clarithromycin, erythromycin, tylosin	Protein synthesis: binds to 50S subunit bacterial ribosome inhibiting translation	Drug efflux, altered drug target, inactivation of drug	23S rRNA genes
Quinolone	Ciprofloxacin, nalidixic acid, norfloxacin	DNA replication: inhibits bacterial DNA gyrase and topoisomerase IV enzyme	Drug efflux, altered target	*gyrA*, *gyrB*, *parC*, *parE*, *grlA*
Rifamycin	Rifampicin	RNA replication: binds to RNA polymerase	Altered target	*rpoB*

Andersson and Hughes (2010); Bryskier (2005); Davies and Davies (2010); Walsh (2003).

Clinical and epidemiological evidence on the effectiveness of stopping antibiotic treatment as a strategy for reducing resistance is both limited and mixed. Clinical studies have shown that in some cases, resistant bacteria remained abundant in the population (Enne et al. 2001; Sundqvist et al. 2010) or even increased in frequency (Arason et al. 2002) despite the absence of drug, while in others the proportion of resistant bacteria within the population declined (Seppala et al. 1997; Austin et al. 1999; Bergman et al. 2004; Gottesman et al. 2009), as expected. In epidemiological studies, reducing the use of antibiotics often leads to a reduction in the frequency of resistant strains, but it rarely succeeds in eliminating them altogether (Salyers and Amabile-Cuevas 1997; Andersson 2003; Enne 2010; Johnsen et al. 2011).

Evidently, the strategy of stopping the use of a drug once resistance has evolved is not always effective at eliminating resistance. The question is why? Three hypotheses could account for the persistence of AMR strains in the absence of antibiotic (Box 1; Andersson 2003; Andersson and Hughes 2010). First, genetic linkage between the resistance gene(s) of interest and selected loci may lead to genetic co-selection (Borrell et al. 2013) and prevent the elimination of resistance. Many instances of persistence in multidrug-resistant strains or plasmid-mediated resistance are likely due to this mechanism. Second, the fitness costs incurred by resistance mutations may be compensated by second-site mutations that increase fitness without compromising resistance. Such compensatory evolution has been observed in both *in vitro* (Levin et al. 2000), *in vivo* (Björkman et al. 2000), and in clinical studies (Björkholm et al. 2001; Nagaev et al. 2001; Gagneux et al. 2006; Comas et al. 2011). Third, the pleiotropic costs of resistance among mutations may be so highly variable as to sometimes include ‘no-cost’ mutations (Sander et al. 2002; Ramadhan and Hegedus 2005), those that have fitness indistinguishable from (or even greater than) their antibiotic-sensitive ancestor in the absence of antibiotic. This last hypothesis has proven challenging to evaluate because we know very little about variation in costs of resistance among different genetic targets. Previous work has shown that costs of resistance among single-step, chromosomal mutations can be highly variable (Kassen and Bataillon 2006), and the literature contains a number of reports of putatively cost-free mutations, including streptomycin resistance in the *rpsL* locus of *Mycobacterium smegmatis* (Sander et al. 2002), isoniazid resistance in *katG* of *Mycobacterium tuberculosis* using a mouse model (Pym et al. 2002) and quinolone resistance in *gyrA* and *parC* of *Streptococcus pneumoniae* (Gillespie et al. 2002).

**Box 1: Mechanisms of gaining and maintaining antibiotic resistance**

Prokaryotic microbes can gain resistance *de novo* by adaptive evolution or via horizontal gene transfer of resistance cassettes between microbes. Resistance can be maintained, in the absence of antibiotic selection, in three ways. Resistance mutations may incur no fitness costs and thus remain in the population in the absence of antibiotic selection pressure. Alternately, costs of resistance can be compensated via second-site mutations that restore organismal fitness in the absence of antibiotic selection. Finally, genetic co-selection can occur whereby there is a genetic linkage between a resistance-conferring gene and either other selected genetic markers or other selected resistance mutations to different antibiotics, thereby enabling nonselected resistance to remain within the population.

To explore the nature of the variation in fitness costs among resistance mutations in more detail, we collate data from the literature on the fitness effects of single chromosomal mutational events that confer antibiotic resistance from a wide range of pathogenic bacterial species. Our objective is to examine the prevalence of so-called ‘no-cost’ resistance mutations with the aim of evaluating whether these could make a substantial contribution to the persistence of AMR (Box 2). We focus on studies that measure fitness directly through competitive assays between a strain with a resistance mutation and the isogenic strain lacking that mutation. This method is preferred over alternatives such as the measurement of population growth rates in pure culture because it is an integrated measure involving all phases of the growth cycle and can capture aspects of competition such as toxin production that may not be reflected in pure culture assays.

**Box 2: Glossary**

Compensatory mutation: A second-site mutation that occurs after a mutation that confers resistance, which lessens or alleviates the fitness costs associated with resistance.

Cross-resistance: The propensity of a genetic change that confers resistance to one drug also to affect resistance to a different drug (by either increasing or decreasing resistance).

Epistasis: When the fitness effect of a mutation is modulated by its interactions with other genes or mutations in the genome.

Genetic co-selection: The occurrence of genetic linkage between the resistance-conferring gene and other selected genetic markers. Thus, even though a nonselected resistance gene might confer a cost, it could remain in the population because of its genetic linkage to a second marker.

Genetic plasticity: The alterable nature of prokaryotic genomes that enables the fluid exchange of DNA from one microorganism to another.

Horizontal gene transfer: The acquisition of a gene by a means other than direct inheritance from a parent cell (vertical transfer). Common in many bacteria and archaea, mechanisms of horizontal gene transfer include transformation, conjugation, and transduction.

Minimum inhibitory concentration: The lowest concentration of an antibiotic that will inhibit the visible growth of a microorganism after overnight incubation.

Relative fitness: the capability of a genotype or individual to survive and reproduce in comparison with a second genotype or individual.

Previous work has highlighted the potential importance of no-cost resistance mutations, and the variation in costs of resistance more generally, in pathogenic bacteria (Andersson 2003, 2006; Andersson and Hughes 2010). To our knowledge, no formal meta-analysis on the relative costs of antibiotic resistance mutations has been performed. In this article, we analyze 179 mutations (121 unique), comprising eight bacterial species and 16 antibiotics, and address the following questions: Are certain antibiotics or species more likely to be associated with no-cost resistance? If so, why? Is there a correlation between the magnitude of the fitness cost and the level of resistance conferred by a given mutation? If higher levels of resistance require a cell to devote more resources to detoxifying or eliminating a drug or involve mutations of greater phenotypic effect, we might expect a negative relationship between MIC and fitness. However, this hypothesis has rarely been tested directly. Answering these questions provides a glimpse into some of the most basic patterns associated with resistance mutations and their effects on fitness, a subject that has received surprisingly little direct attention in the literature.

## Methods

We identified suitable studies to include in our data set by searching the online database Web of Science with the keywords ‘antibiotic resistance’ + ‘fitness cost’ published as of November 2013. Additional studies were found by searching the reference sections of these articles. Many studies could not be included because fitness was measured as the growth rate of each strain rather than via competitive fitness assays.

The principle behind a competitive fitness assay is that a fitter type will outcompete a less fit type when co-cultured in the same set of growth conditions. The rate at which one type excludes the other is a measure of its fitness. Estimating competitive fitness requires monitoring the change in relative frequencies of otherwise isogenic sensitive and resistant strains over time. Different research groups use slightly different methods to calculate fitness, so here we have recalculated all fitness estimates in terms of the Malthusian growth parameter to facilitate direct comparisons (see Box 3).

To be included in our database, an article had to satisfy strict selection criteria: (i) data needed to include an estimate of both mean and variance of competitive fitness, (ii) competitive fitness had to be measured via *in vitro* assays, (iii) resistance had to be conferred by a single mutational event, and (iv) the study needed to be performed in bacteria. We thus rejected many studies that did not include the relevant measures. Altogether, 24 studies were included in the analysis comprising 16 antibiotics (Table S1) from eight antibiotic classes. These studies further included a total of eight bacterial species (Table S2) including *Escherichia coli*, a ubiquitous Gram-negative bacterium, and *S. pneumoniae*, an important Gram-positive opportunistic pathogen. Four papers were excluded because replicate measures of fitness were not provided, making it impossible to estimate the variance in fitness (Billington et al. 1999; Binet and Maurelli 2005; Enne et al. 2005; Nessar et al. 2011). Four further papers were excluded because competitive fitness was calculated *in vivo*, which is not a comparable metric to relative fitness calculated *in vitro* because it lacks a measure of generation time (Nagaev et al. 2001; Gustafsson et al. 2003; Luo et al. 2005; Luangtongkum et al. 2012). Two papers were excluded because relative fitness measures were illustrated graphically, without providing the numerical measures necessary for the meta-analysis (Björkholm et al. 2001; Huitric et al. 2010).

**Box 3: Estimation of competitive fitness**

Competitive assays provide the ‘gold standard’ for measuring fitness. In its simplest form, a competition experiment allows for the estimation of fitness for a focal strain (either a single genotype or a population) relative to a defined, typically ‘wild-type’ competitor, in a given laboratory environment. The focal strain and the wild-type competitor need to be readily distinguishable, for example, via a phenotypic marker (lacZ+ vs lacZ−, or alternative fluorescent markers) or by genotyping. To estimate the costs of antibiotic resistance, the competition environment should be antibiotic-free and is typically a standard laboratory medium such as Lysogeny Broth (LB). The focal strain and the wild type are competed together for a fixed period of time, often 24 h, and samples taken at the beginning and end of the competition allow the researcher to determine the number of focal and wild-type cells in the population. Fitness of the focal strain can then be inferred from the change in its relative abundance: If the focal strain suffers a fitness cost, then its frequency will decrease.

Several formulae have been proposed for estimating the selection coefficient on a genotype, *s*, from competition data (where relative fitness is given by 1 + *s*). Lenski et al. (1991) consider fitness in terms of a Malthusian growth model, where the growth parameter for a strain is the number of doublings that it experiences over a given period of time. As such, the selection coefficient on the focal strain is defined as follows:

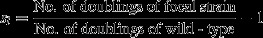

Note that *s*_*l*_ is a unit less parameter.

Alternatively, Dykhuizen and Hartl (1983) estimate the selection coefficient as

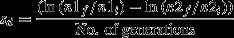

where *n*1_*f*_ and *n*1_*i*_ are the number of cells of the focal strain at the end and the beginning of the assay, and *n*2_*f*_ and *n*2_*i*_ are the number of cells of the wild-type strain at the end and the beginning of the assay. Note that *s*_*d*_ has units generations^−1^.

Both estimates of fitness are widespread in the literature, and we see no principled reason to prefer one to the other. In the context of the current meta-analysis, and more broadly, it is important to know the relationship between these two fitness estimators: To what extent do they agree in terms of the magnitudes of *s*? As such, we simulated pair-wise competitions using a simple growth model, in which each genotype grows according to a Poisson process. Samples were drawn from the simulated competition experiments, and *s*_*l*_ and *s*_*d*_ were estimated from the same data.

**Figure 1:**
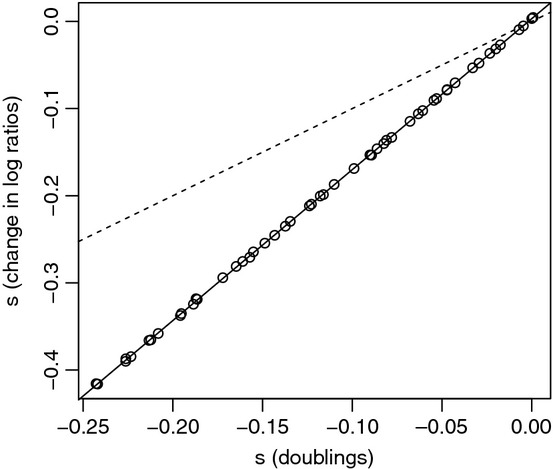
Estimation of selection coefficients using the Lenski and Dykhuisen estimators. Competition experiments were simulated for two strains, with the wild-type strain doubling in each time unit, and the growth rate of the focal strain reduced compared to wild type by 0 to 0.25. Competition lasted six generations, starting with a 50:50 ratio of the two strains and an initial population size of 1 million. Each data point represents the mean values of *s* for 100 simulations. For each replicate, an average of 100 individuals were sampled and used to calculate *s*_*l*_ (*y*-axis) and *s*_*d*_ (*x*-axis). The dashed line represents a 1:1 relationship, and the solid line gives the linear regression of *s*_*l*_ on *s*_*d*_.

Notably, the magnitude of *s*_*l*_ is systematically larger than the magnitude of *s*_*d*_. Figure1 shows the relationship between *s*_*l*_ and *s*_*d*_ for a set of simulations where growth rates of the competing strains varied from 0 to 0.25, with the initial frequency of the focal strain set to 0.5 and competition carried out over six generations. Note that there is a tight linear relationship between *s*_*l*_ and *s*_*d*_, with *s*_*l*_ exceeding *s*_*d*_ by a factor of about 1.7. The slope of the regression line appears to be insensitive to starting frequency and is weakly affected by the number of generations of competition: So long as the competition experiment proceeds for four or more generations, *s*_*l*_ exceeds *s*_*d*_ by a factor of 1.7.

Given that competitive fitness is quantified fairly easily in bacteria and is such an inclusive fitness measure, it was surprising to us that more studies have not employed this method. Many other studies investigating costs of resistance used growth rate as a proxy for fitness, which, as outlined above, incorporates only a single component of bacterial fitness. Including growth rate studies would have increased the sample size of our meta-analysis; however, the inclusion of such data would reduce the clarity of the analysis because measures of growth rate and competitive fitness are poorly correlated. To examine this relationship in more detail for our data set, we examined 35 mutations that supplied estimates of competitive fitness and for which we could compute a relative doubling time (Gillespie et al. 2002; Hurdle et al. 2004; Mariam et al. 2004). The data included 35 different antibiotic resistance mutations from three different bacterial species and four different antibiotics. In these studies, competitive fitness and relative doubling time were not correlated (*r*^2^ = 0.00734, *P* = 0.619). We encourage future studies investigating costs of resistance mutations to use competitive assays to measure the relative fitness of resistant and sensitive strains.

For the purposes of this study, we focused on resistance caused by single mutational events. The rationale behind this is simple: We need to be confident that resistance, and any associated fitness cost, is due to that mutation only and not other, co-occurring mutations. In our study, the vast majority of these mutations are single nucleotide polymorphisms (SNPs) although three small (3, 9, or 13 nucleotides, respectively) deletions were also included. All are chromosomal mutations because our interest is in the effects of these mutations on the fitness of the bacterial genotype itself, not the fitness of a plasmid on which the mutation arises. Thus, we excluded studies that examined resistance gained by plasmids, horizontal gene transfer, those examining multidrug resistance, and studies which examined the fitness costs of multiple resistance mutations (Hurdle et al. 2004) within the same genetic background.

We report measures of drug resistance as the fold-increase in minimum inhibitory concentration (MIC) relative to the drug-sensitive ancestor. If absolute drug concentration was reported, then these data were converted to fold-increase in MIC by dividing the concentration of drug required to inhibit growth in the resistant strain by the concentration of drug required to inhibit growth in the sensitive ancestor. If MICs were reported as a greater than value, the numerical value itself was recorded.

We analyzed the data set using a random effects meta-analysis, using the *metagen* function within the meta package of r (R Development Core Team 2013; Schwarzer 2013). A random effects model is more appropriate than a fixed effects model as we cannot be certain that all studies included in our meta-analysis have equal variances. A relative fitness value >1 indicates the mutation is both resistant and beneficial relative to the isogenic strain lacking the mutation; values <1 indicate the resistant mutation is costly. We evaluated the statistical significance of our fitness estimates by examining the 95% confidence intervals of the mean relative fitness. If these did not overlap with one, the fitness estimate was considered significant. *Q*-statistics were used to examine the heterogeneity of relative fitness values among groups. Each mutational event conferring resistance is taken to be a single unit of analysis, the rationale being that single mutational events occur independently of each other and often in different genetic backgrounds.

## Results

### Are there costs of resistance?

We found a significant fitness cost of resistance mutations (mean fitness = 0.880, *z* = 129.3, *P* < 0.0001). The data exhibited significant total heterogeneity in their response (*Q*_total_ = 22 966, *P* < 0.0001), indicating the presence of further explanatory variables within the data set.

To describe the data in more detail, we plotted the standard error of mean fitness against mean fitness for all mutations in our collection (Figure S1). A linear regression testing for funnel plot asymmetry is significant (*t*_177_ = −4.48, *P* < 0.001), indicating bias in the data toward, in this case, costly mutations. The most parsimonious interpretation is that resistance mutations are often genuinely costly. It seems unlikely that this result represents a publication bias because, if anything, the observation of no-cost mutations is the more novel result. Notably, there is also substantial variation in costs of resistance with at least some mutations exhibiting little or no cost.

### Variation in costs of resistance

**Figure 2:**
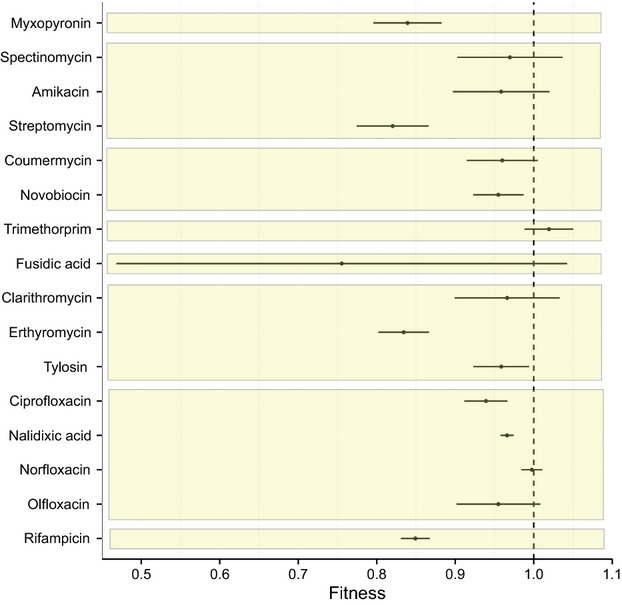
The mean relative fitness and 95% confidence intervals of antibiotic resistance mutations associated with a given antibiotic, grouped by class of antibiotic (from top to bottom alpha-pyrone, aminoglycoside, coumarin, dihydrofolate reductase inhibitor, fusidane, macrolide, quinolone and rifamycin). A fitness value of <1 indicates a fitness cost in the absence of the antibiotic.

**Figure 3:**
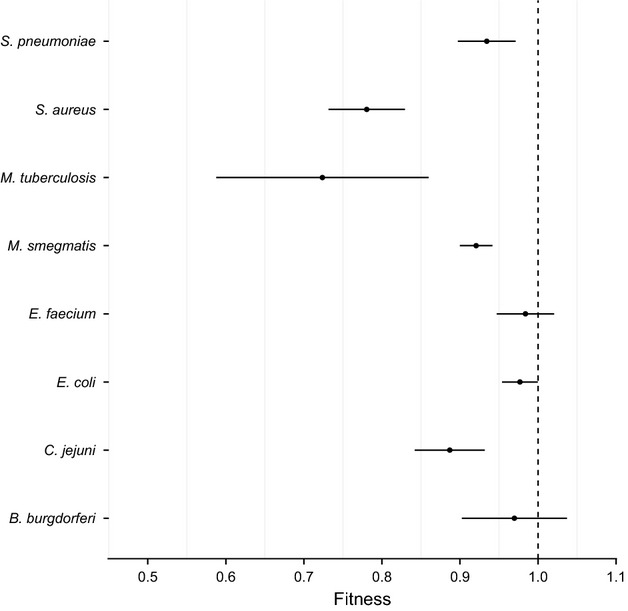
The mean relative fitness and 95% confidence intervals of antibiotic resistance mutations associated with a given species. A fitness value of <1 indicates a fitness cost in the absence of the antibiotic.

Variation in costs of resistance can arise either because some mutations are costly and others are not, irrespective of the genetic background in which they occur, or because a given mutation is not costly in some genetic backgrounds but is costly in others. We investigated these alternatives by repeating our analysis with drug class, drug, or species as explanatory factors. A main effect of species indicates that the fitness effect of a given mutation depends on the genetic background in which it occurs while main effects of either drug or drug class indicate that the mutations themselves differ in their costs, independent of genetic background. We found evidence to support both explanations. There was a significant difference in the fitness costs of resistance mutations between drug classes (Figure S2, *Q*_between_ = 144, *P* < 0.0001), between different drugs (Fig.2, *Q*_between_ = 282, *P* < 0.0001), and between different bacterial species (Fig.3, *Q*_between_ = 75.5, *P* < 0.0001). The mean relative fitness of resistance mutations associated with each of these different subgroups can be seen in Figs3, and S2, respectively. This result must be interpreted with caution, however, because the data set is severely unbalanced. Most resistance mutations are unique to a particular species and drug. Some of the variation is likely attributable to a lack of data, for example, there are only four resistance mutations associated with fusidic acid (Table S1), while other sources of variation might be because there is only a single drug–bacterium comparison for a given species, for example, all eleven mutations measured for *M. tuberculosis* confer resistance to rifampicin (Table S2). Nevertheless, our data suggest that costs of resistance can be highly variable and can depend on the class of drug used, the mutation itself, and the species within which that resistance mutation occurs.

**Figure 4:**
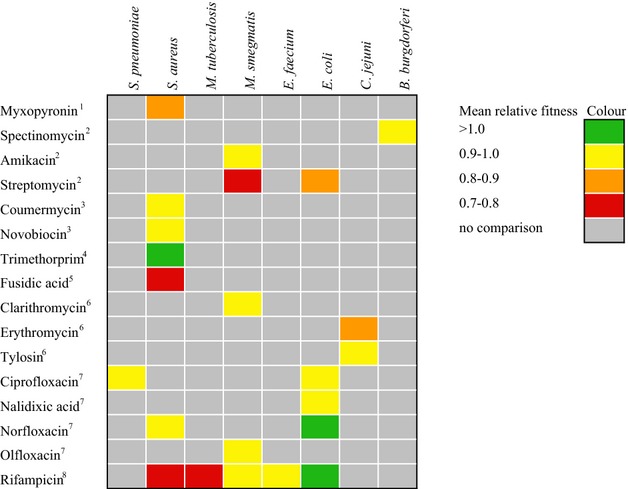
Species–antibiotic comparisons of the mean relative fitness of resistance mutations in the absence of the antibiotic. Numbers indicate antibiotic class: 1 – alpha-pyrone, 2 – aminoglycoside, 3 – coumarin, 4 – dihydrofolate reductase inhibitor (DHRI), 5 – fusidane, 6 – macrolide, 7 – quinolone and 8 – rifamycin.

Notably, some mutations appear to be either genuinely cost-free or the costs are so small they cannot be detected in these assays. In our data set, these putatively ‘no-cost’ mutations are associated with resistance to fusidanes and dihydrofolate reductase inhibitors (Fig.2, Figure S2). Of note, the mean fitness of mutations conferring resistance to the dihydrofolate reductase inhibitor trimethoprim was >1 (Fig.2), indicating that these resistance mutations are beneficial in the absence of drug. Additionally, resistance mutations in two species, *Enterococcus faecium* and *Borrelia burgorferi*, showed no evidence for a cost of resistance on average while those recovered from all other species were on average costly (Fig.3). The average fitness of each species and antibiotic comparison yielded no clear patterns in costs of resistance (Fig.4).

The sparseness of our data set precludes us from doing a fully factorial analysis of mutations, drugs, and species. However, we can perform such an analysis for a subset of our data, namely the resistance mutations associated with quinolone and rifamycin drug classes for both *Staphylococcus aureus* and *E. coli* (Table S3). The simplest meta-analysis shows a significant fitness cost of resistance mutations (mean fitness = 0.874, *z* = 64.4, *P* < 0.0001), with significant heterogeneity (*Q*_total_ = 11592, *P* < 0.0001). The fully factorial linear model that treats species and drug class as fixed effects revealed a significant interaction between drug class and species (*F*_3,80_ = 14.5, *P* < 0.0001), but no significant differences associated with the main effect of species or drug class. While this result is based on a limited data set, it does lend support to the idea that the fitness effect of resistance mutations in the absence of drugs depends on both the drug class and the genetic background in which those mutations appear.

The cell wall is an important target of mutations that confer antibiotic resistance, and thus intuitively, it seems possible that there may be a difference in fitness costs associated with antibiotic resistance between Gram-positive, which has a much thicker layer of peptidoglycan in their cell wall, and Gram-negative bacteria, which has a much thinner cell wall. Gram-positive bacteria had a significantly greater fitness costs associated with resistance mutations (mean fitness = 0.822) when compared with Gram-negative bacteria (mean fitness = 0.973, *t*_156_ = −5.19, *P* < 0.0001). Again, caution must be used in interpreting this result because there were a greater number of Gram-negative than Gram-positive bacteria in our data set.

### The relationship between MIC and cost of resistance

**Figure 5:**
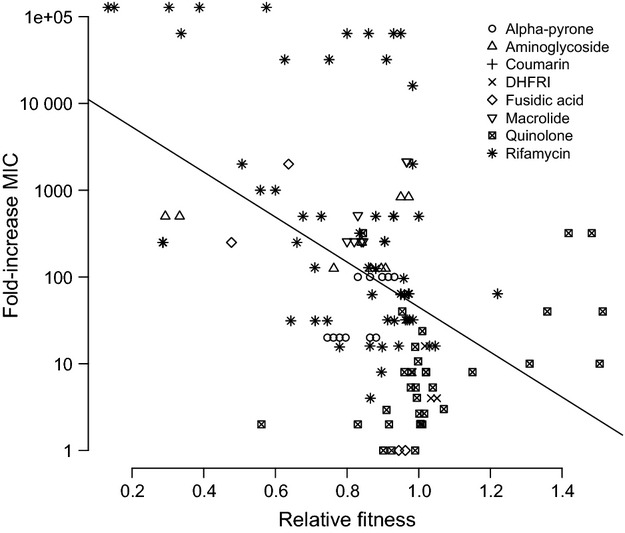
The level of resistance conferred by a mutation is negatively correlated with its’ fitness in the absence of the antibiotic (*r*^2^ = 0.228). Different symbols are associated with different classes of antibiotic.

We regressed MIC against relative fitness to test the prediction that high levels of resistance impose greater fitness costs than lower levels of resistance (Fig.5). The relative fitness of a resistance mutation is negatively correlated with the fold-increase in MIC conferred by the mutation (Fig.5, *t*_126_ = −6.21, *P* < 0.0001), with fold-increase in MIC accounting for 22.8% of the variation in fitness (*F*_1,126_ = 38.6, *P* < 0.0001). A subset of data was used for this analysis because seven studies did not measure MIC (Schrag and Perott 1996; Criswell et al. 2006; Gagneux et al. 2006; Balsalobre and de la Campa 2008; Hao et al. 2009; Trindade et al. 2009; Borrell et al. 2013). Although it would be of interest to perform separate regressions for each class of antibiotic to see if the correlation holds across different mechanisms of action, for many of the drug classes, sample sizes are too small to permit reliable regression coefficients to be estimated.

## Discussion

Using meta-analysis, we have found that most resistance mutations in bacteria confer a fitness cost. This result is not surprising as many antibiotics target important cellular processes and resistance to them either disrupts those processes or imposes large energetic burdens that reduce competitive ability against sensitive strains. However, we have also found that there is substantial variation in fitness costs among species and drugs. This variation is large enough to include, occasionally, what might be classified as no-cost resistance mutations. This class of resistance mutation is not common, at least in our data set, and the actual cost associated with a particular mutation can depend on the genotype in which the mutation occurs. Costs will also likely depend on the environment in which the genotype is growing due to variation in resource identity and abundance, as well as the general level of stress imposed on the cell. We cannot examine this hypothesis in more detail, unfortunately, as the appropriate data are not available. Furthermore, we know little about how well measures of costs *in vitro* correlate to those incurred *in vivo*. Nevertheless based on the available data we do have, we conclude that no-cost resistance mutations are likely not major contributors to persistent drug resistance in the absence of antibiotic.

Can we move from a phenomenological description of the variation in costs to a more mechanistic interpretation? Why, for example, are resistance mutations costly in some situations and not in others? Clearly, epistasis can play a role. In an evolutionary context, epistasis refers to a situation in which the fitness effect of a mutation depends on its genetic background. For example, ciprofloxacin resistance in *Campylobacter jejuni* is often via mutations in *gyrA*, which encodes a DNA gyrase. In one case, a resistance mutation (C257T) that showed a fitness benefit in one strain of *C. jejuni* was costly in another strain (Luo et al. 2005). Environment can also be important. The fitness estimates we examined here are all obtained *in vitro* and may not always reflect key aspects of fitness *in vivo*. Indeed, at least two resistance mutations in *fusA* that each confer resistance to fusidic acid in *Salmonella typhimurium* (Macvanin et al. 2003, 2004) and two mutations in *rpoB* (S464P and S531L) conferring rifampicin resistance in *S. aureus* show evidence that fitness costs change depending on whether it is measured *in vitro* or *in vivo* (Yu et al. 2005; Gagneux et al. 2006).

Further generalizations are difficult because our data set is quite sparse. Most of the fitness estimates for a given mutation are gathered in single environments, which makes generalization difficult. Moreover, the fitness cost of the same resistance mutation is rarely assayed in more than one strain, let alone more than one species. Thus, our ability to draw strong inferences on the causes of such variation in costs of resistance remains limited.

Nevertheless, at least two results appear noteworthy and warrant further investigation. The first is that there is fairly good evidence that mutations that confer larger MICs are more costly. This result is in line with previous studies that found a similar relationship between MIC and growth rate (Ender et al. 2004; Hurdle et al. 2004). It has also been shown that the first mutations that arise and confer resistance to ciprofloxacin in *Pseudomonas aeruginosa* are generally costly (Wong et al. 2012). This relationship can be understood very generally in terms of Fisher's geometric model of adaptation: Mutations of large effect for one phenotype (MIC) have pleiotropic effects on other phenotypes, the result being that an individual is knocked off a local fitness peak. More mechanistically, the causes of pleiotropy due to resistance mutations stem either from the fact that dealing with high levels of toxin in the environment is an energetically costly process that takes resources away from other cellular functions, or because resistance is gained via mutations that alter or disrupt enzyme function and the production of essential proteins.

The second notable result is that the presence of a thicker cell wall, one of the defining features of Gram-positive bacteria, is associated with larger fitness costs. While this result must be interpreted with caution because it is based on few data points, it is interesting that it holds for many different kinds of resistance mutation, including those that confer resistance through both small molecule efflux and target binding. It thus appears to be a very general result although the biological reason for this remains unclear. One, rather simplistic, suggestion is that the presence of a cell wall imposes an additional energetic burden on toxin clearance that Gram-negative bacteria do not have to deal with.

The main clinical implication of this work is that no-cost mutations are probably not a common reason why antibiotic resistance persists in the absence of drug use. Rather, it seems much more likely that persistence is due either to co-selection of genetically linked mutations or because the fitness cost of resistance mutations is often compensated by mutations elsewhere in the genome. Indeed, previous work suggests that compensatory mutations can arise within a few generations following the emergence of resistance (Björkman et al. 2000; Maisnier-Patin et al. 2002; Kugelberg et al. 2005; Paulander et al. 2007; Bataillon et al. 2011; Sousa et al. 2012; Wong et al. 2012; de Vos et al. 2013). Furthermore, the presence of additional resistance mutations can compensate for the cost of an initial resistance mutation, a form of positive epistasis for fitness (Trindade et al. 2009).

That said there is some evidence that resistance mutations with low or no fitness costs can be prevalent in clinical populations. For example, the spectrum of mutations in *rpoB* that cause rifampicin resistance in clinical isolates of *M. tuberculosis* and *S. aureus* is biased in favor of low-cost mutations (O'Sullivan et al. 2005; O'Neill et al. 2006). The K424R substitution in the 30S ribosomal protein S12 also does not exhibit a fitness cost associated with resistance in both *S. typhimurium* and *M. smegmatis*, and this same mutation is also primarily responsible for resistance to streptomycin in clinical isolates of *M. tuberculosis* (Böttger et al. 1998; Sander et al. 2002). Other mutations in *rpoB* can be costly, as evidenced by the fact that putative compensatory mutations in *rpoA* and *rpoC* are routinely isolated alongside some resistance-causing *rpoB* mutations (Comas et al. 2011). Thus, while no-cost mutations may not be a general explanation for why antibiotic resistance persists in the absence of drug, it may be important in specific cases.

Taken together, these observations suggest that no-cost mutations cannot be automatically dismissed as an explanation for why antibiotic resistance persists in clinical settings even after the offending drug is removed from use. Whether costs of resistance are an effective guide to predicting the prevalence of resistance following reduced drug prescription remains an open question. Enne (2010) investigated this question directly and found mixed results, with reduced prescriptions leading to reduced prevalence in some cases but not others. Notably, in two cases where our results indicate a significant cost of resistance for a given bacterial species, Enne also found that prevalence was reduced following prescription reduction of penicillin for *S. pneumoniae* (Austin et al. 1999) and quinolones for *E. coli* (Gottesman et al. 2009), lending some support to the predictive ability of costs of resistance from individual mutations. However, other examples show contrasting results. For example, persistent quinolone resistant *E. coli* was found in a remote community where quinolones were not prescribed (Pallecchi et al. 2012), suggesting co-selection. It has been suggested that, in the case of quinolone resistance in this species, co-selection could occur because resistance can be plasmid-mediated (Wang et al. 2003).

The take-home message here is that there may not be any simple connection between the cost of resistance for individual mutations and clinical prevalence of resistance. Given the variety of factors that can modulate costs – epistasis between the resistance mutation and genetic background or even other resistance mutations, the environment, the occurrence of compensatory mutations, and genetic linkage between the resistance mutation and other mutations under selection – this should not be surprising. The evolution of costs of resistance and its connection to clinical treatment is a more complex issue than was initially thought. Future work should focus on disentangling the contributions of these various factors, in particular compensatory mutations that seem to evolve very quickly alongside costly resistance mutations, to the persistence of antibiotic-resistant strains in clinical and environmental settings.

## Data accessibility

Data for this study are available from the Dryad Digital Repository: http://doi.org/10.5061/dryad.5rc47.

## Supporting Information

**Figure S1.** Funnel plot of mean relative fitness plotted against standard error.

**Figure S2.** The mean relative fitness and 95% confidence intervals of antibiotic resistance mutations associated with a given antibiotic class.

**Table S1.** Studies included in analysis, indicating antibiotic used.

**Table S2.** Studies included in analysis, indicating bacterial species used.

**Table S3.** Studies used for subgroup analyses by bacterial species and drug class.
