# Supplementary figures and images for: The fitness costs of antibiotic resistance mutations
Source: Evol Appl. 2014 Aug 27;8(3):273–83. doi: 10.1111/eva.12196 (PMC4380921; doi:10.1111/eva.12196)

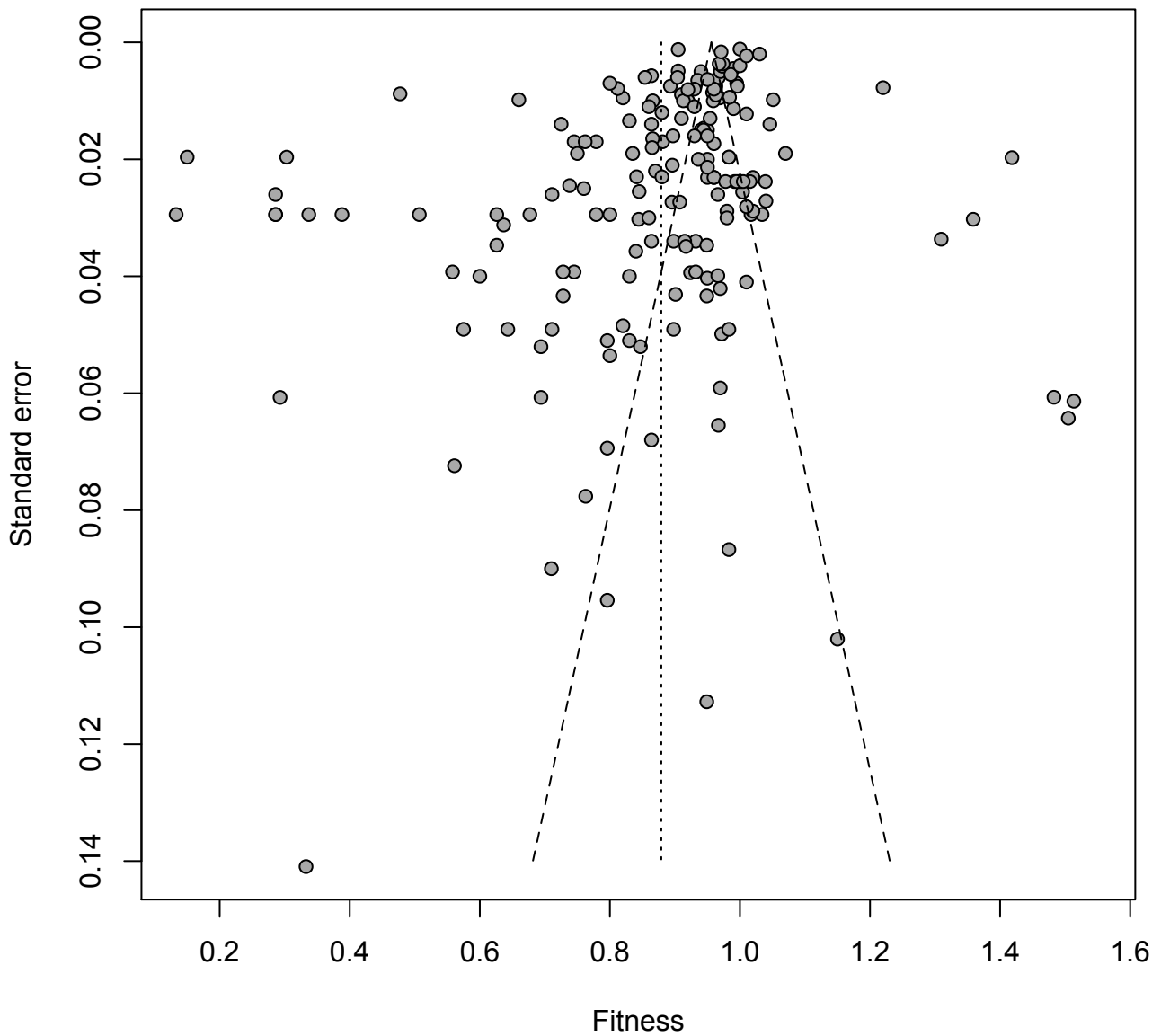

Supplement: Supplementary file 1 — Figure S1. Funnel plot of mean relative fitness plotted against standard error. [file eva0008-0273-sd1.pdf]

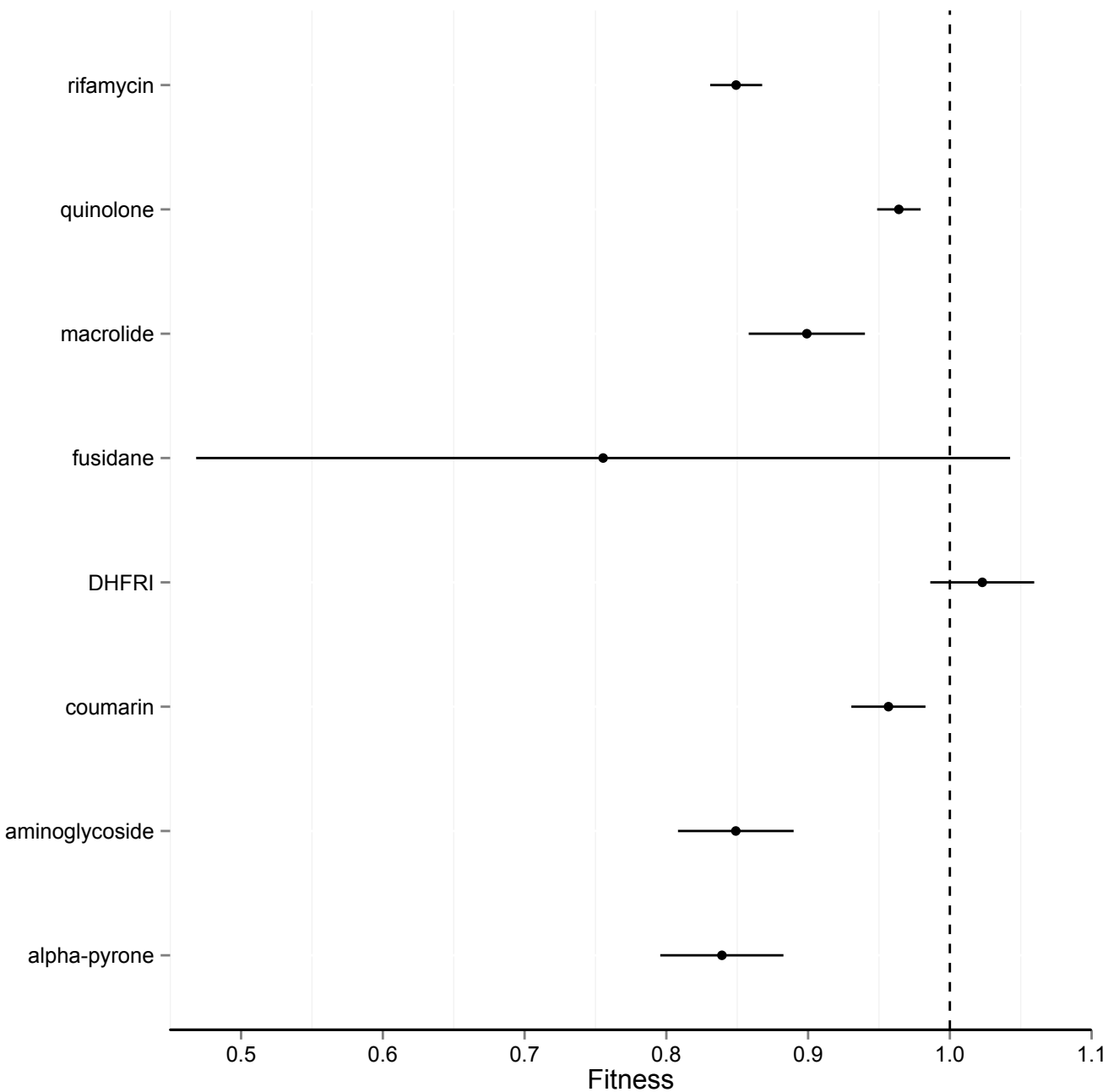

Supplement: Supplementary file 2 — Figure S2. The mean relative fitness and 95% confidence intervals of antibiotic resistance mutations associated with a given antibiotic class. [file eva0008-0273-sd2.pdf]
