# Supplementary material for: The fitness costs of antibiotic resistance mutations
Source: Evol Appl. 2014 Aug 27;8(3):273–83. doi: 10.1111/eva.12196 (PMC4380921; doi:10.1111/eva.12196)
Supplement: Supplementary file 3 — Table S1. Studies included in analysis, indicating antibiotic used. [file eva0008-0273-sd3.doc]

Supplementary Table 1. Studies included in analysis, indicating antibiotic used.

| Study | No. of mutations | amikacin | ciprofloxacin | clarithromycin | coumermycin | erthryomycin | fusidic acid | myxopyronin | nalidixic acid | norfloxacin | novobiocin | olfloxacin | rifampicin | spectinomycin | streptomycin | trimethorprim | tylosin |
| --- | --- | --- | --- | --- | --- | --- | --- | --- | --- | --- | --- | --- | --- | --- | --- | --- | --- |
| Almofti et al. 2011 | 1 |  |  |  |  | X |  |  |  |  |  |  |  |  |  |  |  |
| Balsalobre & de la Campa 2008 | 5 |  | X |  |  |  |  |  |  |  |  |  |  |  |  |  |  |
| Besier et al. 2005 | 4 |  |  |  |  |  | X |  |  |  |  |  |  |  |  |  |  |
| Borrell et al. 2013 | 9 |  |  |  |  |  |  |  |  |  |  | X | X |  |  |  |  |
| Criswell et al. 2006 | 2 |  |  |  |  |  |  |  |  |  |  |  |  | X |  |  |  |
| Enne et al. 2004 | 9 |  |  |  |  |  |  |  |  |  |  |  | X |  |  |  |  |
| Gagneux et al. 2006 | 9 |  |  |  |  |  |  |  |  |  |  |  | X |  |  |  |  |
| Gillespie et al. 2002 | 1 |  | X |  |  |  |  |  |  |  |  |  |  |  |  |  |  |
| Han et al 2009 | 4 |  |  |  |  | X |  |  |  |  |  |  |  |  |  |  |  |
| Hao et al. 2009 | 5 |  |  |  |  |  |  |  |  |  |  |  |  |  |  |  | X |
| Lindgren et al. 2005 | 8 |  |  |  |  |  |  |  |  | X |  |  |  |  |  |  |  |
| Marcusson et al. 2009 | 5 |  | X |  |  |  |  |  |  |  |  |  |  |  |  |  |  |
| Mariam et al. 2004 | 3 |  |  |  |  |  |  |  |  |  |  |  | X |  |  |  |  |
| O’Neill et al. 2006 | 22 |  |  |  |  |  |  |  |  |  |  |  | X |  |  |  |  |
| Reynolds 2000 | 9 |  |  |  |  |  |  |  |  |  |  |  | X |  |  |  |  |
| Rodriguez-Verdugo et al. 2013 | 8 |  |  |  |  |  |  |  |  |  |  |  | X |  |  |  |  |
| Rozen et al. 2007 | 7 |  | X |  |  |  |  |  |  |  |  |  |  |  |  |  |  |
| Sander et al. 2002 | 11 | X |  | X |  |  |  |  |  |  |  |  |  |  | X |  |  |
| Schrag & Perrot 1996 | 2 |  |  |  |  |  |  |  |  |  |  |  |  |  | X |  |  |
| Srivastava et al. 2012 | 11 |  |  |  |  |  |  | X |  |  |  |  |  |  |  |  |  |
| Trinidade et al. 2009 | 19 |  |  |  |  |  |  |  | X |  |  |  | X |  | X |  |  |
| Vickers et al. 2009 | 4 |  |  |  |  |  |  |  |  |  |  |  |  |  |  | X |  |
| Vickers et al. 2007 | 7 |  |  |  | X |  |  |  |  | X | X |  |  |  |  |  |  |
| Wichelhaus et al. 2002 | 14 |  |  |  |  |  |  |  |  |  |  |  | X |  |  |  |  |

Almofti, Y. A., M. Dai, Y. Sun, H. Hao, Z. Liu, G. Cheng, and Z. Yuan

2001. The physiologic and phenotypic alterations due to macrolide

exposure in Campylobacter jejuni. International Journal of Food

14 Microbiology 151:52–61.

Besier, S., A. Ludwig, V. Brade, and T. A. Wichelhaus 2005. Compensa-

tory adaptation to the loss of biological fitness associated with acquisi-

tion of fusidic acid resistance in Staphylococcus aureus. Antimicrobial

Agents and Chemotherapy 49:1426–1431.

Enne, V. I., A. A. Delsol, J. M. Roe, and P. M. Bennett 2004. Rifampicin

resistance and its fitness cost in Enterococcus faecium. The Journal of

16 antimicrobial chemotherapy 53:203–207.

Han, F., S. Pu, F. Wang, J. Meng, and B. Ge 2009. Fitness cost of macro-

lide resistance in Campylobacter jejuni. International Journal of Anti-

17 microbial Agents 34:462–466.

Lindgren, P. K., L. L. Marcusson, D. Sandvang, N. Frimodt-Moller, and

D. Hughes 2005. Biological cost of single and multiple norfloxacin

resistance mutations in Escherichia coli implicated in urinary tract

infections. Antimicrobial Agents and Chemotherapy 49:2343–2351.

Marcusson, L. L., N. Frimodt-Moller, and D. Hughes 2009. Interplay in

the selection of fluoroquinolone resistance and bacterial fitness. PLoS

Pathogens 5:e10000541.

Reynolds, M. G. 2000. Compensatory evolution in rifampin-resistant

23 Escherichia coli. Genetics 156:1471–1481.

Rodriguez-Verdugo, A., B. S. Gault, and O. Tenaillon 2013. Evolution of

Escherichia coli rifampicin resistance in an antibiotic-free environment

24 during thermal stress. BMC Evolutionary Biology 13:50.

Rozen, D. E., L. McGee, B. R. Levin, and K. P. Klugman 2007. Fitness

costs of fluoroquinolone resistance in Streptococcus pneumoniae. Anti-

25 microbial Agents and Chemotherapy 51:412–416.

Srivastava, A., D. Degen, Y. W. Ebright, and R. H. Ebright 2012. Fre-

quency, spectrum, and nonzero fitness costs of resistance to myxopy-

ronin in Staphylococcus aureus. Antimicrobial Agents and

27 Chemotherapy 56:6250–6255.

Vickers, A. A., A. J. O’Neill, and I. Chopra 2007. Emergence and mainte-

nance of resistance to fluoroquinolones and coumarins in Staphylococ-

cus aureus: predictions from in vitro studies. The Journal of

Antimicrobial Chemotherapy 60:269–273. 28

Vickers, A. A., N. J. Potter, C. W. G. Fishwick, I. Chopra, and A. J.

O’Neill 2009. Analysis of mutational resistance to trimethoprim in

Staphylococcus aureus by genetic and structural modelling techniques.

Antimicrobial Agents and Chemotherapy 63:1112–1117.

Wichelhaus, T. A., B. Boddinghaus, S. Besier, V. Schafer, , Brade V, and

A. Ludwig. 2002. Biological cost of rifampin resistance from the per-

spective of Staphylococcus aureus. Antimicrobial Agents and Chemo-

therapy 46:3381–3385.
