# Supplementary material for: The fitness costs of antibiotic resistance mutations
Source: Evol Appl. 2014 Aug 27;8(3):273–83. doi: 10.1111/eva.12196 (PMC4380921; doi:10.1111/eva.12196)
Supplement: Supplementary file 4 — Table S2. Studies included in analysis, indicating bacterial species used. [file eva0008-0273-sd4.doc]

Supplementary Table 2. Studies included in analysis, indicating bacterial species used.

| Study | No. of mutations | *B. burgdorferi* | *C. jejuni* | *E. coli* | *E. faecium* | *M. smegmatis* | *M. tuberculosis* | *S. aureus* | *S. pneumoniae* |
| --- | --- | --- | --- | --- | --- | --- | --- | --- | --- |
| Almofti et al. 2011 | 1 |  | X |  |  |  |  |  |  |
| Balsalobre & de la Campa 2008 | 5 |  |  |  |  |  |  |  | X |
| Borrell et al. 2013 | 9 |  |  |  |  | X |  |  |  |
| Besier et al. 2005 | 4 |  |  |  |  |  |  | X |  |
| Criswell et al. 2006 | 2 | X |  |  |  |  |  |  |  |
| Enne et al. 2004 | 9 |  |  |  | X |  |  |  |  |
| Gagneux et al. 2006 | 9 |  |  |  |  |  | X |  |  |
| Gillespie et al. 2002 | 1 |  |  |  |  |  |  |  | X |
| Han et al 2009 | 4 |  | X |  |  |  |  |  |  |
| Hao et al. 2009 | 5 |  | X |  |  |  |  |  |  |
| Lindgren et al. 2005 | 8 |  |  | X |  |  |  |  |  |
| Marcusson et al. 2009 | 5 |  |  | X |  |  |  |  |  |
| Mariam et al. 2004 | 3 |  |  |  |  |  | X |  |  |
| O’Neill et al. 2006 | 22 |  |  |  |  |  |  | X |  |
| Reynolds 2000 | 9 |  |  | X |  |  |  |  |  |
| Rodriguez-Verdugo et al. 2013 | 8 |  |  | X |  |  |  |  |  |
| Rozen et al. 2007 | 7 |  |  |  |  |  |  |  | X |
| Sander et al. 2002 | 11 |  |  |  |  | X |  |  |  |
| Schrag & Perrot 1996 | 2 |  |  | X |  |  |  |  |  |
| Srivastava et al. 2012 | 11 |  |  |  |  |  |  | X |  |
| Trinidade et al. 2009 | 19 |  |  | X |  |  |  |  |  |
| Vickers et al. 2009 | 4 |  |  |  |  |  |  | X |  |
| Vickers et al. 2007 | 7 |  |  |  |  |  |  | X |  |
| Wichelhaus et al. 2002 | 14 |  |  |  |  |  |  | X |  |
