# Supplementary material for: The fitness costs of antibiotic resistance mutations
Source: Evol Appl. 2014 Aug 27;8(3):273–83. doi: 10.1111/eva.12196 (PMC4380921; doi:10.1111/eva.12196)
Supplement: Supplementary file 5 — Table S3. Studies used for subgroup analyses by bacterial species and drug class. [file eva0008-0273-sd5.doc]

Supplementary Table 3. Studies used for subgroup analyses by bacterial species and drug class.

| Study | No. of mutations | *S. aureus* quinolone | *S. aureus* rifamycin | *E. coli* quinolone | *E. coli* rifamycin |
| --- | --- | --- | --- | --- | --- |
| Balsalobre & de la Campa 2008 | 5 |  |  | X |  |
| Lindgren et al. 2005 | 8 |  |  | X |  |
| O’Neill et al. 2006 | 22 |  | X |  |  |
| Rodriguez-Verdugo et al. 2013 | 8 |  |  | X |  |
| Sander et al. 2002 | 9 |  |  |  | X |
| Trinidade et al. 2009 | 14 |  |  |  | X |
| Vickers et al. 2007 | 4 | X |  |  |  |
| Wichelhaus et al. 2002 | 14 |  | X |  |  |
